# Geschlechtersensible Gesundheitsindikatoren für die Gesundheitsberichterstattung am Robert Koch-Institut (GBE-GI) – Ein Pilotprojekt im Rahmen der „Joint Action PreventNCD“

**DOI:** 10.1007/s00103-024-03959-6

**Published:** 2024-10-02

**Authors:** Hande Gencer, Anke-Christine Saß, Franziska Prütz

**Affiliations:** Abteilung für Epidemiologie und Gesundheitsmonitoring, Fachgebiet 24 Gesundheitsberichterstattung, Robert Koch-Institut, General-Pape-Straße 62–66, 12101 Berlin, Deutschland

**Keywords:** Geschlechtersensibilität, Gesundheitsberichterstattung, Gesundheitsindikatoren, Scoping-Review, Delphi-Studie, Gender sensitivity, Health reporting, Health indicators, Scoping review, Delphi study

## Abstract

Eine auf geschlechtersensiblen Indikatoren basierende Gesundheitsberichterstattung (GBE) kann Aufschluss über Geschlechterungleichheiten geben, die Gesundheitsförderung und Gesundheitsversorgung verbessern, *Gender-Mainstreaming* fördern sowie relevante Informationen für Gleichstellungsmaßnahmen zur Verfügung stellen. Bisher gibt es kein Set von geschlechtersensiblen Gesundheitsindikatoren für die Gesundheitsberichterstattung des Bundes am Robert Koch-Institut. Im Rahmen der *Joint Action Prevent Non-Communicable Diseases* sollen in einem Pilotprojekt geschlechtersensible Gesundheitsindikatoren identifiziert, systematisch bewertet und in die Webseite für Gesundheitsberichterstattung des Robert Koch-Instituts integriert werden. Dies geschieht in 3 Arbeitspaketen: In einem Scoping-Review wird zunächst die Evidenzlage zu geschlechtersensiblen Gesundheitsindikatoren sowie zu theoretischen Erklärungsansätzen für Geschlechterungleichheit in EU- und OECD-Mitgliedsstaaten ab 2014 durchleuchtet (Arbeitspaket 1). Darauf aufbauend werden die identifizierten Indikatoren und Erklärungsansätze aufbereitet und in einem strukturierten Konsensprozess im Rahmen eines 3‑stufigen Delphi-Verfahrens bestehend aus einer Fokusgruppendiskussion und einem Online-Survey bewertet und ausgewählt (Arbeitspaket 2). Der systematisch bewertete Indikatorensatz wird schließlich in die Webseite für Gesundheitsberichterstattung des Robert Koch-Instituts integriert (Arbeitspaket 3). Die Ergebnisse werden über Fachpublikationen und Konferenzbeiträge disseminiert.

**Zusatzmaterial online:**

Zusätzliche Informationen sind in der Online-Version dieses Artikels (10.1007/s00103-024-03959-6) enthalten.

## Abstract (en)

Health reporting (GBE) based on gender-sensitive indicators can provide information on gender inequalities, improve health promotion and healthcare, promote gender mainstreaming, and provide relevant information for gender equality measures. To date, there is no set of gender-sensitive health indicators for Federal Health Reporting at the Robert Koch Institute. As part of the *Joint Action Prevent Non-Communicable Diseases*, gender-sensitive health indicators are to be identified, evaluated, and integrated into the Robert Koch Institute’s health reporting website in a pilot project. This is done in three work packages. In a scoping review, the available evidence on gender-sensitive health indicators and theoretical explanations for gender inequality in EU and OECD member states since 2014 will initially be assessed (work package 1). Building on this, identified indicators and explanatory approaches will be collated for evaluation and selection in a structured consensus process as part of a three-stage Delphi approach consisting of a focus group discussion and an online survey (work package 2). The evaluated indicator set will then be integrated into the Robert Koch Institute’s health reporting website (work package 3). The results will be disseminated via scientific publications and conference presentations. An English full-text version of this article is available at SpingerLink as Supplementary Information.

## Hintergrund

Geschlecht ist sowohl in seiner biologischen Dimension „Sex“ als auch der soziokulturellen Dimension „Gender“ [1] eine wichtige Determinante der Gesundheit, entlang derer Gesundheitsoutcomes und -risiken zwischen Frauen, Männern sowie genderdiversen Personen ungleich verteilt sind [2, 3]. Allgemein wird Geschlechterungleichheit in internationalen Erhebungen in unterschiedlicher Form routinemäßig gemessen, z. B. mittels *Social Institutions and Gender Index* der OECD [4], *Gender Inequality Index* der UNDP^1^ sowie mittels *Gender Equality Index* der Europäischen Union (EU)^2^. Die kontinuierliche Erhebung und Bereitstellung geschlechtersensibler Gesundheitsdaten im Rahmen von Gesundheitsmonitoring und Gesundheitsberichterstattung sind von großer Public-Health-Relevanz: Im Sinne der Strategie *Health in All Policies *können sie die Gesundheitsförderung und Gesundheitsversorgung verbessern, *Gender-Mainstreaming* fördern und relevante Informationen für Gleichstellungsmaßnahmen zur Verfügung stellen [5–7]. Dies erfordert jedoch die Entwicklung von geschlechtersensiblen Gesundheitsindikatoren.

Gesundheitsindikatoren sind ein wichtiger Bestandteil der Gesundheitsberichterstattung des Bundes (GBE). Sie sind definiert als quantitative oder qualitative Messgrößen, die Informationen mit Public-Health-Relevanz, etwa zu Bevölkerungsgesundheit, Gesundheitsdeterminanten sowie Gesundheitsversorgung, bereitstellen. Sie liefern vergleichbare und handlungsrelevante Informationen über Trends und Veränderungen des Gesundheitszustands der Bevölkerung in oder zwischen unterschiedlichen Settings (z. B. Länder, Regionen, Gemeinden, Bevölkerungsgruppen) [8]. Gesundheitsindikatoren müssen die psychometrischen Anforderungen der Validität und Reliabilität erfüllen; sie müssen in der Lage sein, Veränderungen im Zeitverlauf zu messen, sie müssen einfach zu nutzen, verständlich und ethisch vertretbar sein [8, 9].

Geschlechtersensible Indikatoren geben Aufschluss über die Gesundheitssituation von Frauen und Männern. Sie haben zudem das Potenzial, die gesundheitliche Lage und die Bedürfnisse von Personen abzubilden, die intersexuell, nichtbinär und nichtcisgeschlechtlich (sich nicht mit dem ihnen bei Geburt zugeordneten Geschlecht identifizierend) sind [10, 11]. Ein reiner Geschlechtervergleich der gesundheitlichen Lage von Frauen und Männern birgt die Gefahr, ein zu homogenes Bild zu zeichnen und Geschlechterklischees zu verfestigen [9]. Eine geschlechtersensible GBE kann dieser Gefahr entgegenwirken; sie bietet die Chance, durch den Einbezug von soziokulturellen Faktoren auch Entwicklungen von Gendernormen und Genderrollen nachzuzeichnen [6].

### Geschlechterunterschiede in der Gesundheit

Die Gesundheitsberichterstattung am Robert-Koch-Institut (RKI) [12] stützt sich auf Leitlinien für die Einbeziehung der Geschlechterperspektive in die GBE [13, 14]. Dies geschieht zum einen dadurch, dass Ergebnisse nach Geschlecht differenziert werden [15], und zum anderen durch eigene Berichte zu Geschlecht und Gesundheit [2, 3, 16].

Diese Art der Gesundheitsberichterstattung ermöglicht es, Geschlechterunterschiede in der Gesundheit aufzuzeigen, die unter anderem am Beispiel von nichtübertragbaren Krankheiten (NCDs) deutlich werden: Herz-Kreislauf-Erkrankungen, Krebserkrankungen und Atemwegserkrankungen sind die häufigste Todesursache in Deutschland^3^ und weltweit [17]. Im Jahr 2023 waren bei Frauen 36 % und bei Männern 32 % aller Todesfälle auf eine Herz-Kreislauf-Erkrankung zurückzuführen, gefolgt von Krebserkrankungen (22 % bei Frauen, 25 % bei Männern) und Krankheiten des Atmungssystems (7 % bei Frauen, 8 % bei Männern)^3^. Dennoch gelten Herz-Kreislauf-Erkrankungen überwiegend als „männliche Erkrankung“ und Frauen unterschätzen häufig ihr Erkrankungsrisiko [18]. Geschlechterunterschiede zeichnen sich auch im Gesundheitsverhalten ab. Frauen verhalten sich häufig gesundheitsbewusster als Männer: Sie rauchen seltener täglich oder stark, trinken weniger Alkohol und ernähren sich ausgewogener als Männer [2].

An der Schnittstelle zwischen Geschlecht und weiteren sozialen Differenzkategorien wird deutlich, dass Gesundheitsunterschiede nicht nur zwischen, sondern auch innerhalb der Geschlechtergruppen bestehen. Das zeigt sich insbesondere bei NCDs: Sozial benachteiligte Personen, wie Menschen mit niedrigem Bildungsniveau, geringem Einkommen oder prekärer Beschäftigung, haben im Vergleich zu sozial bessergestellten Personen ein höheres Erkrankungs- und Sterberisiko [19, 20]. So sind sozial benachteiligte Frauen und Männer häufiger von Herzinfarkten [21], Schlaganfällen [22], chronischer Bronchitis [19] und Diabetes [20] betroffen als Personen mit einem höheren sozioökonomischen Status. Auch bei Krebserkrankungen zeigen sich für Männer und Frauen soziale Ungleichheiten bei der Inzidenz bestimmter Krebsarten. So kommen Magen- und Gebärmutterhalskrebs häufiger unter sozial benachteiligten Frauen vor, wohingegen sozial bessergestellte Frauen häufiger von Brust- und Hautkrebs betroffen sind [23]. Beim Gesundheitsverhalten zeigen sich ebenfalls Unterschiede: Laut GEDA 2019/2020-EHIS [18] sind Frauen mit niedriger Bildung seltener sportlich aktiv, rauchen häufiger und essen seltener Obst und/oder Gemüse als Frauen mit hoher Bildung, welche wiederum häufiger riskante Trinkmengen an Alkohol konsumieren.

Menschen mit Migrationsgeschichte stellen eine heterogene Gruppe dar, sie unterscheiden sich sowohl nach soziodemografischen als auch nach migrationsbezogenen Merkmalen. Dementsprechend variieren die Krankheitsrisiken und Gesundheitschancen auch innerhalb der Gruppe der Frauen und Männer mit Migrationsgeschichte stark [18]. Die Datenlage zu diesen Gruppen ist jedoch unzureichend. Einzelne Auswertungen, wie die des Sozio-oekonomischen Panels (SOEP) für das Jahr 2016 [2], zeigen beispielsweise, dass Frauen mit Migrationsgeschichte zwar häufiger chronische Beschwerden aufweisen, jedoch seltener von bestimmten NCDs, wie Herzerkrankungen, chronischen Rückenbeschwerden und Diabetes, betroffen sind als Frauen ohne Migrationsgeschichte. Insbesondere zugewanderte Frauen sind seltener sportlich aktiv, rauchen seltener und trinken seltener Alkohol in riskanten Mengen als Frauen ohne Zuwanderungsgeschichte, wobei es Unterschiede nach Migrationsgeschichte und Herkunftsland gibt [24, 25].

### Geschlechtersensibilität in der Gesundheitsberichtserstattung (GBE)

Geschlechtersensible Gesundheitsindikatoren zielen darauf ab, geschlechtsrelevante gesundheitliche Ungleichheiten und Geschlechterungleichheit als soziale Determinanten von Gesundheit zu beleuchten. Sie werden verwendet, um Ungleichheiten in Bezug auf Gesundheit und gesundheitsbezogene Outcomes zwischen Frauen, Männern, genderdiversen Personen sowie deren Subgruppen zu messen [6]. Mittels geschlechtersensibler Indikatoren können gesundheitliche Outcomes und Phänomene mit den ihnen zugrunde liegenden soziokulturellen Normen und soziostrukturellen Machtsystemen (z. B. Geschlechternormen, Heteronormativität, Rassismus und Ableismus) in Beziehung gesetzt werden. Sie können Aufschluss über die Trends von Geschlechterungleichheit in der Gesundheit geben [26].

Angelehnt an das Modell von der WHO und UNAIDS [26] lassen sich folgende Kategorien der Geschlechtersensibilität von Gesundheitsindikatoren unterscheiden:

1. *geschlechterspezifische Indikatoren*, die sich auf ein einzelnes Geschlecht oder eine Gruppe von Personen mit bestimmten biologischen Merkmalen beziehen (z. B. die Prävalenz von Prostatakrebs oder Endometriose),
2. *geschlechterbezogene Indikatoren*, die sich auf eine Geschlechtsgruppe beziehen (z. B. die Prävalenz von Krebserkrankungen oder NCDs für eine Geschlechtsgruppe),
3. *geschlechterdifferenzierte Indikatoren*, die Geschlechtsunterschiede in Bezug auf weitere soziodemografische Variablen messen (z. B. die Prävalenz von Krebserkrankungen oder NCDs nach Geschlecht und Altersgruppe, Migrationsgeschichte oder Haushaltseinkommen),
4. *Indikatoren für Geschlechterungleichheit*, welche Ungleichheiten zwischen Geschlechtern messen oder repräsentieren (z. B. Indikatoren, die plausible Zusammenhänge zwischen Gesundheitsoutcomes und soziostrukturellen/normativen Gegebenheiten herstellen, wie *Gender-Pay-Gap, Gender-Care-Gap* oder der Anteil von Alleinerziehenden in der Bevölkerung).

Erklärungsansätze für Geschlechterunterschiede in der Gesundheit und damit zusammenhängenden Faktoren können dazu beitragen, dass die GBE differenzierter über Gesundheitsoutcomes und -risiken berichten kann. Häufig fehlen diese jedoch; gerade hier besteht Forschungsbedarf [9, 11, 27]. Die Einbeziehung von theoretischen Rahmenmodellen und Ansätzen zur Erklärung geschlechterspezifischer Ungleichheiten in der Gesundheit kann bei der Entwicklung von Gesundheitsindikatoren zu einem besseren Verständnis von Gesundheit und gesundheitsbezogenen Einflussfaktoren führen [5]. Die Genderanalyse ist ein theoretischer Ansatz, der Sex/Gender als zentrale Analysekategorie versteht. Sie zielt darauf ab, geschlechtsbezogene Ungleichheiten vor dem Hintergrund verschiedener individueller, sozialer und struktureller Umstände zu identifizieren und zu thematisieren, die Personen in den ihnen zugewiesenen Geschlechterrollen unterschiedlich beeinflussen [28]. Eine intersektional ausgerichtete Genderanalyse verwendet Sex/Gender als Hauptanalyseachse und hebt ihre Überschneidung mit anderen Kategorien sozialer Differenzierung (z. B. sozioökonomische, soziokulturelle und soziodemografische Faktoren) hervor [29, 30]. Geschlecht als Gesundheitsdeterminante wirkt sich je nach sozialer Verortung unterschiedlich aus, was durch ineinandergreifende Privilegierungs- und Benachteiligungssysteme (z. B. Rassismus, Behindertenfeindlichkeit, (Hetero‑)Sexismus, *Ageism* und Klassismus) beeinflusst wird [31, 32]. Um eine adäquatere Darstellung der gesundheitlichen Situation bestimmter Bevölkerungsgruppen sowie eine bessere Einbeziehung gesellschaftlicher (Macht‑)Verhältnisse und Strukturen als Gesundheitsdeterminanten zu ermöglichen, ist es daher hilfreich, intersektional informierte theoretische Ansätze in die GBE einzubeziehen [6].

### Projektziele

Diese sozialen und geschlechterbezogenen gesundheitlichen Ungleichheiten unterstreichen die Relevanz einer geschlechtersensiblen und intersektional informierten GBE. Bislang fehlt jedoch ein Set an geschlechtersensiblen Gesundheitsindikatoren, um Geschlechtersensibilität in der GBE zu stärken. Hier setzt das Pilotprojekt zu geschlechtersensiblen Gesundheitsindikatoren für die GBE (GBE-GI) zur Stärkung der Prävention von NCDs, inklusive Krebserkrankungen, als Teil der *Joint Action Prevent Non-Communicable Diseases* (JA PreventNCD, 2024–2027, https://www.preventncd.eu/, siehe Infobox 1) an, das von der Europäischen Union im Rahmen des EU4Health-Programms (GA – 101128023) kofinanziert wird.

Das Pilotprojekt zielt darauf ab, geschlechterspezifische Bedarfe für die Krebs- und NCD-Prävention zu identifizieren und durch einen intersektionalen Ansatz weiter zu spezifizieren. Dabei soll auf die Arbeit des vom Bundesministerium für Bildung und Forschung geförderten Projekts *AdvanceGender* (2017–2021; [6]) und auf den RKI-Bericht „Gesundheitliche Lage der Frauen in Deutschland“ (2020; [2]) aufgebaut werden. In diesem Pilotprojekt werden bestehende geschlechtersensible Indikatoren/Indikatorensätze zusammengefasst, bewertet und zu einem Kernset von Indikatoren zusammengestellt, die geschlechterspezifische gesundheitliche Ungleichheiten und Determinanten für eine kontinuierliche GBE mit dem Fokus auf NCD- und Krebsprävention darstellen sollen. Dazu gehören zum Beispiel auch häufige gynäkologische Erkrankungen wie Endometriose und Uterusmyome sowie das Thema sexuelle Gesundheit. Durch den intersektionalen Ansatz können weitere Ungleichheitsdimensionen berücksichtigt werden, was eine präzisere Ausrichtung der Indikatoren auf tiefer gegliederte gefährdete Gruppen ermöglicht. Auf dieser Basis können Ansätze zur geschlechtersensiblen Förderung von Gesundheitsressourcen und Risikominderung in ausgewählten Zielgruppen entwickelt werden. Die Projektziele samt der Arbeitspakete (AP) umfassen im Einzelnen:

- Identifizierung relevanter geschlechtersensibler Gesundheitsindikatoren für die GBE sowie theoretischer Erklärungsansätze für geschlechterspezifische gesundheitliche Ungleichheiten (AP1: Scoping-Review),
- Entwicklung und Bewertung eines Indikatorensatzes geschlechtersensibler Gesundheitsindikatoren auf der Basis eines theoretischen Rahmenmodells und anschließende Zusammenfassung zu einem Kernsatz an Indikatoren (AP2: Delphi-Verfahren),
- Integration des Kernsatzes geschlechtersensibler Gesundheitsindikatoren in die Webseite für Gesundheitsberichterstattung des RKI (AP3).

## Methoden

### Arbeitspaket 1: Bestandsaufnahme bestehender Indikatoren(sätze)

Die systematische Bestandsaufnahme existierender geschlechtersensibler Gesundheitsindikatoren erfolgt über eine systematische Literaturrecherche in Form eines Scoping-Reviews. Neben den Indikatoren werden auch Erklärungsansätze und theoretische Modelle für Geschlechterungleichheit in der Gesundheit, die der Auswahl von Indikatoren(sätzen) der eingeschlossenen Literatur zugrunde liegen, identifiziert. Hierfür wird eine Datenbanksuche in den elektronischen Datenbanken Medline, PsycInfo, Embase, Scopus und CINAHL im Zeitraum zwischen 2014 und 2024 durchgeführt. Die Suchstrategie wird mittels englischsprachiger Suchbegriffe und *Medical Subject Headings* (MeSH-Terms) entlang der 3 Themen Geschlechtersensibilität („gender sensitivity“), Gesundheitsberichterstattung („health reporting“) sowie Gesundheitsindikatoren („health indicators“) abgeleitet. Zusätzlich werden die Referenzlisten identifizierter Volltexte nach weiteren geeigneten Publikationen durchsucht (*Backward Citation Searching*). Außerdem erfolgt eine Suche nationaler und internationaler Webseiten mit Bezug zu Gesundheitsmonitoring und Gesundheitsberichterstattung sowie eine Internetsuche auf Google. Die Studie beschränkt sich auf die Settings der EU-27 und OECD-Mitgliedstaaten, was Vorteile in Bezug auf die Relevanz, Datenqualität und Handhabbarkeit der Ergebnisse hat sowie mit einer besseren Anwendbarkeit auf die GBE in Deutschland einhergeht. Gleichzeitig bringt das Eingrenzen der Studie auf diese Settings auch Nachteile mit sich, insbesondere hinsichtlich einer fehlenden globalen Perspektive. Die eingeschlossenen Publikationsformate beinhalten neben wissenschaftlichen Fachartikeln jeglichen Studiendesigns auch Berichte, Broschüren, Buchkapitel und Webseiten. Eine ausführliche Darstellung der methodischen Vorgehensweise ist in einem Studienprotokoll dokumentiert (10.17605/OSF.IO/SHR8M).

### Arbeitspaket 2: Systematische Bewertung und Auswahl der Indikatoren

Auf Grundlage der strukturierten Bestandsaufnahme werden die Indikatoren aufbereitet und in einem strukturierten Konsensprozess in Rahmen eines 3‑stufigen Delphi-Verfahrens bewertet und ausgewählt. Das methodische Vorgehen orientiert sich dabei an den Vorarbeiten der RKI-Projekte *Improving Health Monitoring in Old Age* (IMOA; [33]) sowie *Diabetes-Surveillance in Deutschland* [34]. Die Aufbereitung des Indikatorensatzes für das Delphi-Verfahren samt der Definition von Handlungsfeldern und Themenbereichen innerhalb dieser Handlungsfelder orientiert sich an dem theoretischen Rahmenmodell geschlechtsrelevanter Determinanten gesundheitlicher Ungleichheit, das auf Grundlage der Ergebnisse aus Arbeitspaket 1 entwickelt werden soll.

Die Identifikation von Teilnehmenden für den Expert:innen-Workshop erfolgt durch bestehende Kontakte und ein Schneeballsystem sowie durch ein strukturiertes Stakeholder-Mapping, bei dem relevante Stakeholder aus den Bereichen Public Health, Intersektionalitäts- und Genderforschung identifiziert werden sollen. Um möglichst partizipativ vorzugehen, ist zudem der gezielte Kontakt zu Verbänden, die zu diesem Thema arbeiten, und weiteren Akteur:innen geplant.

In einer ersten Stufe werden identifizierte Stakeholder:innen per E‑Mail kontaktiert und erhalten einen standardisierten Bewertungsbogen in Form einer Online-Befragung. Die einzelnen Indikatoren werden durch die Stakeholder:innen entlang vordefinierter Handlungsfelder und Themenbereiche auf einer 9‑stufigen Relevanzskala von 1 = geringe Relevanz bis 9 = hohe Relevanz bewertet [33]. Zusätzlich werden Anmerkungen und Fragen der Teilnehmenden zu den Indikatoren in Freitextfeldern erhoben. Der Bewertungsmaßstab für die Einschätzung der Relevanz orientiert sich u. a. an dem im Scoping-Review identifizierten theoretischen Rahmenmodell.

In der zweiten Stufe des Delphi-Verfahrens werden ca. 15 Expert:innen zu einem Workshop eingeladen. Im Rahmen des Expert:innen-Workshops werden die Ergebnisse der Online-Befragung vorgestellt und diskutiert. Anschließend erhalten die Teilnehmenden einen schriftlichen Bewertungsbogen für Indikatoren, die in der ersten Bewertungsstufe als hoch relevant oder relevant eingestuft wurden.

In einer dritten und finalen Stufe werden die in der zweiten Bewertungsstufe als hoch relevant oder relevant bewerteten Indikatoren aufbereitet und erneut in Form eines Bewertungsbogens per E‑Mail an alle Stakeholder:innen verschickt. Auch hier wird die Bewertung der Indikatoren auf derselben 9‑stufigen Relevanzskala erfolgen. Außerdem werden qualitatives Feedback und Änderungsvorschläge der Teilnehmenden erhoben.

### Arbeitspaket 3: Integration des Indikatorensatzes in die Webseite für Gesundheitsberichterstattung des Robert Koch-Instituts

Die Ergebnisse des Delphi-Verfahrens werden für die Integration des Indikatorensatzes in die Webseite für Gesundheitsberichterstattung des RKI (www.gbe.rki.de), die voraussichtlich Ende 2024 online geht, in enger Zusammenarbeit mit Expert:innen am RKI aufbereitet. Die Webseite für Gesundheitsberichterstattung des RKI stellt zuverlässige und aktuelle Daten und Informationen zur gesundheitlichen Lage der Bevölkerung in Deutschland zur Verfügung. Sie richtet sich primär an Fachöffentlichkeit und Gesundheitspolitik, aber auch an die allgemeine interessierte Öffentlichkeit. Der Schwerpunkt liegt auf NCDs (z. B. Diabetes, Herz-Kreislauf-Erkrankungen, Krebserkrankungen und psychische Störungen) sowie auf Einflussfaktoren auf die Gesundheit samt sozialer Faktoren und das Gesundheitsverhalten der Menschen. Darüber hinaus werden auch Rahmenbedingungen und die Gesundheitsversorgung berücksichtigt. Außerdem ermöglicht die Webseite einen Einblick in ausgewählte Gesundheitsindikatoren, die interaktiv visualisiert werden und nach Themen und Lebensphasen gefiltert werden können. Zu den Datenquellen, die für die Darstellung der Indikatoren genutzt werden, gehören Gesundheitsstudien des RKI, Daten der amtlichen Statistik, Routinedaten der gesetzlichen Krankenversicherung und Registerdaten. Wichtig dabei ist, dass die Daten repräsentativ für die Bevölkerung sind. Sie sollten außerdem regelmäßig vorliegen und die jeweiligen Kennzahlen zuverlässig abbilden.

## Fazit

Die Ergebnisse des Pilotprojekts zu geschlechtersensiblen Gesundheitsindikatoren in der GBE sollen zu einer geschlechtersensiblen und theoretisch informierten Weiterentwicklung der GBE hinsichtlich der Prävention von NCDs und Krebserkrankungen bei Frauen, Männern und genderdiversen Personen beitragen. Die Einbindung einer analytischen Geschlechterperspektive, die über das geschlechterdifferenzierte Berichten entlang einer binären Sex/Gender-Variable hinausgeht und weitere soziale Differenzkategorien an der Schnittstelle zu Sex/Gender berücksichtigt, kann eine differenziertere Berücksichtigung von Geschlechterungleichheiten in der GBE ermöglichen. Sie eröffnet die Chance, Entwicklungen im Verständnis von Geschlecht und Geschlechterrollen sowie die Diversität zwischen und innerhalb von Geschlechtergruppen besser abzubilden. Eine geschlechtersensible GBE wirkt sich im Sinne der Strategie *Health in All Policies* schließlich auch auf Prävention, Gesundheitsförderung und (gesundheits-)politische Maßnahmen aus und kann zu mehr Geschlechtergerechtigkeit in allen Politikbereichen beitragen.

### Infobox 1 Über die Joint Action Prevent Non-Communicable Diseases (JA PreventNCD)

Die JA PreventNCD (https://www.preventncd.eu/) ist eine von der EU geförderte Initiative, an der insgesamt 25 Länder (EU-Mitgliedstaaten sowie Norwegen, Island und die Ukraine) beteiligt sind. Das gemeinsame Ziel des Verbundprojekts ist die Stärkung der Prävention von NCDs und Krebserkrankungen sowie die Verringerung der damit verbundenen Krankheitslast. Der Schwerpunkt liegt auf der Implementierung wirksamer Strategien und politischer Maßnahmen im Kontext gesellschaftlicher Risikofaktoren. In Deutschland beteiligen sich neben dem RKI als affiliiertes Mitglied unter der Federführung der Bundeszentrale für gesundheitliche Aufklärung (BZgA) zudem das Leibniz-Institut für Präventionsforschung und Epidemiologie (BIPS), das Max Rubner-Institut (MRI) sowie die Medizinische Hochschule Hannover (MHH). Das Pilotprojekt „Gendersensible Gesundheitsindikatoren für die GBE“ ist Teil des Arbeitspakets 7 „Gesundheitliche Ungleichheit“, welches zur Verringerung der gesundheitlichen Ungleichheiten bei NCDs und Krebserkrankungen in Europa beitragen soll. In 5 Aufgabenbereichen beschäftigen sich Mitwirkende des Arbeitspakets 7 u. a. mit der Evidenzsynthese von Ungleichheiten bei NCDs und Krebserkrankungen und deren Risikofaktoren sowie von (gesundheits-)politischen Maßnahmen, um ebendiese Ungleichheiten abzubauen. Außerdem werden Pilotprojekte durchgeführt, die sich in unterschiedlichem Maß mit sozialen Determinanten der Gesundheit sowie assoziierten Risikofaktoren beschäftigen. In dem letzteren Arbeitsbereich ist auch das vorliegende Pilotprojekt angesiedelt.

## Supplementary Information

English translation of the article

## Footnotes

1 https://hdr.undp.org/data-center/thematic-composite-indices/gender-inequality-index#/indicies/GII (zuletzt aufgerufen: 20.08.2024).

2 https://eige.europa.eu/gender-equality-index/2022/domain/health (zuletzt aufgerufen: 20.08.2024).

3 https://www.destatis.de/DE/Themen/Gesellschaft-Umwelt/Gesundheit/Todesursachen/Tabellen/gestorbene_anzahl.html (zuletzt aufgerufen: 04.09.2024).
