# Supplementary material for: Gender-sensitive health indicators for health reporting at the Robert Koch Institute (GBE-GI)—a pilot project as part of the Joint Action PreventNCD project
Source: Bundesgesundheitsblatt Gesundheitsforschung Gesundheitsschutz. 2024 Oct 2;67(11):1315–20. [Article in German] doi: 10.1007/s00103-024-03959-6 (PMC11549133; doi:10.1007/s00103-024-03959-6)
Supplement: Supplementary file 1 — English translation of the article [file 103_2024_3959_MOESM1_ESM.pdf]

This is an English translation of the article: "Geschlechtersensible Gesundheitsindikatoren für die Gesundheitsberichterstattung am Robert Koch-Institut – Ein Pilotprojekt im Rahmen der JA PreventNCD" (Bundesgesundheitsblatt 11/2024). The responsibility for the translation lies solely with the authors. Please note that only the original German-language article can be cited.

Gender-sensitive health indicators for health reporting at the Robert Koch Institute (GBE-GI) – A pilot project as part of the Joint Action PreventNCD project

Hande Gencer[1], Anke-Christine Saß[1], Franziska Prütz[1]

[1] Department of Epidemiology and Health Monitoring, Unit of Health Reporting, Robert Koch Institute, Berlin, Germany

Corresponding author:

Hande Gencer, MA
Robert Koch Institute
General-Pape-Straße 62-66
12101 Berlin
Germany
GencerH@rki.de

ABSTRACT

Health reporting based on gender-sensitive indicators can provide information on gender inequalities, improve health promotion and health care, promote gender mainstreaming and provide relevant information for gender equality measures. To date, there is no set of gender-sensitive health indicators for Federal Health Reporting at the Robert Koch Institute. As part of the *Joint Action Prevent Non-Communicable Diseases*, gender-sensitive health indicators are to be identified, evaluated and integrated into the Robert Koch Institute's health reporting website in a pilot project. This is done in three work packages: In a scoping review, the available evidence on gender-sensitive health indicators and theoretical explanations for gender inequality in EU and OECD member states since 2014 will initially be assessed (work package 1). Building on this, identified indicators and explanatory approaches will be collated for evaluation and selection in a structured consensus process as part of a three-stage Delphi approach consisting of a focus group discussion and an online survey (work package 2). The evaluated indicator set will then be integrated into the Robert Koch Institute's health reporting website (work package 3). The results will be disseminated via scientific publications and conference presentations.

Keywords: gender sensitivity, health reporting, health indicators, scoping review, Delphi study

BACKGROUND

Gender, both in its biological dimension "sex" and the sociocultural dimension "gender" [1], is an important determinant of health, along which health outcomes and risks are unequally distributed between women, men and gender-diverse persons [2, 3]. In general, gender inequality is routinely measured in various forms in international surveys, e.g. using the OECD's *Social Institutions and Gender Index* [4], the *UNDP's Gender Inequality Index*[1] and the European Union's (EU) *Gender Equality Index*[2]. The continuous collection and provision of gender-sensitive health data as part of health monitoring and health reporting is of great relevance to public health: In line with the *Health in All Policies* strategy, it can improve health promotion and health care, promote gender mainstreaming and provide relevant information for gender equality measures [5-7]. This, however, requires the development of gender-sensitive health indicators.

Health indicators are important components of German Federal Health Reporting (GBE). They are defined as quantitative or qualitative metrics that provide information with public health relevance, for example on population health, health determinants and health care. They provide comparable and actionable information on trends and changes in the health status of the population within or between different settings (e.g. countries, regions, municipalities, population groups) [8]. Health indicators must meet the psychometric requirements of validity and reliability; they must be able to measure changes over time, they must be easy to use, understandable and ethical [8, 9].

Gender-sensitive indicators provide information about the health situation of women and men. They also have the potential to illustrate the health situation and needs of are non-cisgender (persons, who do not identify with the gender assigned to them at birth), intersex or non-binary [10, 11]. A mere gender comparison of the health situation of women and men bears the risk of painting too homogeneous a picture and reinforcing gender stereotypes [9]. Gender-sensitive GBE can mitigate this risk; it also offers the opportunity to trace trends in gender norms and gender roles by incorporating socio-cultural factors [6].

Gender differences in health

Health reporting at the Robert Koch Institute (RKI) [12] is based on guidelines for the inclusion of the gender perspective in GBE [13, 14]. This is achieved one the one hand by differentiating results according to gender [15] and on the other hand by means of special reports on gender and health [2, 3, 16].

This type of health reporting allows gender differences in health to be highlighted, for example in the case of non-communicable diseases (NCDs): Cardiovascular diseases, cancers and respiratory diseases are the most common causes of death in Germany[3] and worldwide [17]. In 2023, 36% of all deaths in women and 32% in men were due to cardiovascular disease, followed by cancers (22% in women, 25% in men) and diseases of the respiratory system (7% in women, 8% in men)[3]. Despite this, cardiovascular diseases are predominantly considered a "male disease" and women often underestimate their risk of developing them [18]. Gender differences are also evident in health behavior. Women often adopt more health-conscious behavior than men: they smoke less daily or heavily, drink less alcohol and have a more balanced diet than men [2].

---

[1] https://hdr.undp.org/data-center/thematic-composite-indices/gender-inequality-index#/indicies/GII (Last accessed: 20.08.2024)
[2] https://eige.europa.eu/gender-equality-index/2022/domain/health (Last accessed: 20.08.2024)
[3] https://www.destatis.de/DE/Themen/Gesellschaft-Umwelt/Gesundheit/Todesursachen/Tabellen/gestorbene_anzahl.html (Last accessed: 04.09.2024)

At the intersection between gender and other categories of social differentiation, it shows that health differences exist not only between but also within gender groups. This is particularly evident in the case of NCDs: socially disadvantaged people, such as those with a low level of education, low income or precarious employment, have a higher risk of disease and dying compared to people with higher socio-economic status [19, 20]. Socially disadvantaged women and men are more frequently affected by heart attacks [21], strokes [22], chronic bronchitis [19] and diabetes [20] than people with a higher socio-economic status. When it comes to cancer, there are also social inequalities in the incidence of certain types of cancer for men and women. For example, stomach and cervical cancer are more common among socially disadvantaged women, whereas socioeconomically better-off women are more frequently affected by breast and skin cancer [23]. There are also differences in health behavior: according to GEDA 2019/2020-EHIS data [18], women with a low level of education are less likely to be physically active, smoke more often and eat less fruits and/or vegetables than women with a high level of education, who in turn consume risky amounts of alcohol more often.

People with a migration history represent a heterogeneous group, they differ in terms of both socio-demographic and migration-related characteristics. Accordingly, the disease risks and health opportunities vary greatly within the group of women and men with a migration history [18]. However, the data available on these groups is insufficient. Individual evaluations, such as that of the Socio-Economic Panel (SOEP) for 2016 [2], show, for example, that women with a migration history have more chronic complaints, but are less frequently affected by certain NCDs, such as heart disease, chronic back problems and diabetes, than women without a migration history. Immigrant women in particular are less physically active, less likely to smoke and less likely to drink alcohol in risky quantities than nonimmigrant women, although there are differences according to migration history and country of origin [24, 25].

Gender-sensitivity in health reporting (GBE)

Gender-sensitive health indicators aim to shed light on gender-relevant health inequalities and gender inequality as social determinants of health. They are employed to measure inequalities in health and health-related outcomes between women, men, gender-diverse persons and their subgroups [6]. Gender-sensitive indicators can be used to relate health outcomes and phenomena to the underlying socio-cultural norms and socio-structural power systems (e.g. gender norms, heteronormativity, racism and ableism). They can provide information on the trends of gender inequality in health [26].

Drawing on the WHO & UNAIDS model [26], the following categories of gender sensitivity of health indicators can be distinguished:

1. *Gender-specific indicators* that relate to a single gender or a group of persons with certain biological characteristics (e.g. the prevalence of prostate cancer or endometriosis),
2. *Gender-related indicators* that refer to a gender group (e.g. the prevalence of cancer or NCDs for a gender group),
3. *Gender-differentiated indicators* that measure gender differences in relation to other socio-demographic variables (e.g. the prevalence of cancer or NCDs by gender and age group, migration history or household income),
4. *Gender inequality indicators* that measure or are proxy for gender inequalities (e.g. indicators that establish a plausible link between health outcomes and socio-structural/normative circumstances, such as the gender pay gap, the gender care gap or the proportion of single parents in the population).

Explanatory approaches for gender differences in health and related factors can help GBE to report on health outcomes and risks in a more differentiated way. However, these are often lacking; this is precisely where research is needed [27, 9, 11]. The inclusion of theoretical frameworks and approaches to explain gender inequalities in health can lead to a better understanding of health and health-related determinants when developing health indicators [5]. Gender analysis is a theoretical approach that understands sex/gender as a central category of analysis. It aims to identify and address gender-related inequalities against the background of various individual, social and structural circumstances that influence persons differently in the gender roles assigned to them [28]. An intersectionality-informed gender analysis uses sex/gender as the main axis of analysis and emphasizes its intersection with other categories of social differentiation (e.g. socio-economic, socio-cultural and socio-demographic factors) [29, 30]. Gender as a determinant of health has different effects depending on social location, which is influenced by interlocking systems of privilege and disadvantage (e.g. racism, ableism, (hetero)sexism, ageism and classism) [31, 32]. It is therefore helpful to incorporate intersectionality-informed theoretical approaches into GBE in order to enable a more adequate representation of the health situation of certain population groups and a better inclusion of social (power) relations and structures as determinants of health [6].

Objectives

These social and gender-related health inequalities underline the relevance of gender-sensitive and intersectionality-informed health reporting. To date, however, a set of gender-sensitive health indicators to strengthen gender sensitivity in GBE has been lacking.

This issue is addressed by the pilot project on Gender-Sensitive Health Indicators for GBE (GBE-GI) to promote the prevention of NCDs including cancer as part of the *Joint Action Prevent Non-Communicable Diseases* (JA PreventNCD, 2024-2027, https://www.preventncd.eu/, see Infobox 1), which is co-funded by the European Union as part of the EU4Health program (GA - 101128023).

Infobox 1. About the Joint Action Prevent Non-Communicable Diseases (JA PreventNCD)

The JA PreventNCD (https://preventncd.eu/) is a project co-funded by the EU involving a total of 25 countries (EU Member States, Norway and Ukraine). The overall goal of the joint action is to strengthen the prevention of NCDs and cancer as well as to reduce the associated burden of disease. The focus lies on the implementation of effective strategies and political measures in the context of social risk factors. In Germany, the Leibniz Institute for Prevention Research and Epidemiology (BIPS), the Max Rubner Institute (MRI) and the Hannover Medical School (MHH) are participating alongside the RKI as an affiliated member under the leadership of the Federal Centre for Health Education (BZgA). The pilot project "Gender-sensitive health indicators for the GBE" is part of work package 7 "Health inequalities", which aims to contribute to the reduction of health inequalities in NCDs and cancer in Europe. In five tasks, participants in work package 7 deal with, among other things, the evidence synthesis of inequalities in NCDs and cancer and their risk factors as well as (health) policy measures to reduce these inequalities. In addition, pilot projects are being carried out that deal to varying degrees with social determinants of health and associated risk factors. This pilot project is also located in the latter task.

The pilot aims to identify gender-specific needs for cancer and NCD prevention and to further specify them through an intersectional approach. It builds on the work of the *AdvanceGender* project funded by the Federal Ministry of Education and Research (2017-2021) [6] and the RKI report "Women's Health in Germany" (2020) [2]. In this pilot, existing gender-sensitive indicators/indicator sets will be synthesized, assessed and compiled into a core set of indicators that represent gender inequalities in health and their determinants for continuous health reporting on NCDs and cancer. This includes, for example, common gynecological diseases such as endometriosis and uterine fibroids as well as the topic of sexual health. The intersectionality-informed approach allows further dimensions of inequality to be considered and enables a more precise focus of health indicators on more finely structured risk groups. This provides a basis for developing approaches for the gender-sensitive promotion of health resources and risk reduction in selected target groups. Project objectives and work packages (WP) include the following:

- identification of relevant gender-sensitive health indicators for GBE and theoretical frameworks for gender inequalities in health (WP1: Scoping Review)
- development and assessment of a indicator set of gender-sensitive health indicators based on a theoretical framework model and subsequent summary into a core set of indicators (WP2: Delphi method)
- integration of the core set of gender-sensitive health indicators into the RKI's health reporting website (WP3)

METHODS

Work package 1: Review of existing (sets of) indicators

The systematic search for existing gender-sensitive health indicators is carried out by means of a scoping review study. In addition to the indicators, potential explanatory approaches and theoretical models for gender inequality in health, on which the selection of indicators (sets) from the included literature will be based on, are also identified. For this purpose, a database search is conducted in the electronic databases Medline, PsycInfo, Embase, Scopus and CINAHL in the period between 2014 and 2024. The search strategy is derived using English-language search terms and Medical Subject Headings (MeSH-Terms) along the three topics of gender sensitivity, health reporting and health indicators. In addition, the reference lists of identified full texts are searched for further suitable publications (backward citation searching). Furthermore, a search of national and international websites related to health monitoring and health reporting as well as an internet search on Google is carried out. The study is limited to the settings of the EU-27 and OECD member states, which offers the benefits of relevance, data quality and manageability of the results, as well as better applicability to GBE in Germany. At the same time, limiting the study to these settings also brings disadvantages, particularly with regard to the lack of a global perspective. Publication types will include research articles of all study designs as well as reports, brochures, book chapters and websites. A detailed description of the methodological approach is documented in a study protocol (https://doi.org/10.17605/OSF.IO/SHR8M).

Work package 2: Systematic assessment and selection of indicators

The indicators are prepared on the basis of the scoping review and assessed and selected in a structured consensus process as part of a three-stage Delphi method. The methodological approach is based on the preliminary work of the RKI projects *Improving Health Monitoring in Old Age* (IMOA) [33] and *Diabetes Surveillance in Germany* [34]. The preparation of the indicator set for the Delphi process,

including the definition of action fields and subject areas within them, is based on the theoretical framework model of gender-relevant determinants of health inequality, which is to be developed on the basis of the results from work package 1.

Participants for the expert workshop will be identified through existing contacts and a snowballing system, as well as through structured stakeholder mapping, in which relevant stakeholders from the fields of public health, intersectionality and gender research will be identified. In order to achieve the highest possible level of participation, organizations that deal with this topic and other stakeholders will also be contacted.

In the first stage, identified stakeholders are contacted by email and receive a standardized assessment questionnaire in the form of an online survey. The individual indicators are to be assessed by the stakeholders along predefined action fields and subject areas on a 9-point relevance scale from 1 = low relevance to 9 = high relevance [33]. In addition, comments and questions from the participants on the indicators are to be collected in open text fields. The assessment criteria for the relevance assessment are based, among other things, on the theoretical framework model identified in the scoping review.

In the second stage of the Delphi process, around 15 experts are invited to a workshop. The results of the online survey will be presented and discussed at the expert workshop. The participants will then receive a written assessment sheet for indicators that were classified as highly relevant or relevant in the first assessment stage.

In a third and final stage, the indicators rated as highly relevant or relevant in the second assessment stage are prepared and again sent to all stakeholders by email in the form of an assessment questionnaire. Here, too, the indicators are to be assessed on the same 9-point relevance scale along with qualitative feedback and suggestions for modification from the participants.

Work package 3: Integration of the indicator set into the website for health reporting of the Robert Koch Institute

The results of the Delphi process will be prepared for the integration of the indicator set into the RKI's health reporting website (www.gbe.rki.de), which is expected to go online at the end of 2024, in close cooperation with experts at the RKI. The RKI's health reporting website provides reliable and up-to-date data and information on the health situation of the population in Germany. It is primarily aimed at experts and health policy-makers, but also at the general public. The focus is placed on NCDs (e.g. diabetes, cardiovascular diseases, cancer and mental disorders) as well as factors influencing health, including social factors and people's health behavior. In addition, general environmental factors and healthcare are also considered. The website also provides an insight into selected health indicators, which are visualized interactively and can be filtered by topic and life phase. The data sources used to present the indicators include health studies by the RKI, data from official statistics, routine data from the statutory health insurance system and registry data. It is important that the data is representative of the population. They should also be available on a regular basis and provide a reliable representation of the respective indicators.

CONCLUSION

The results of the pilot project on gender-sensitive health indicators in GBE are intended to contribute to gender-sensitive and theoretically-informed further development of health reporting with regard to the prevention of NCDs and cancer in women, men and gender-diverse persons. The integration of an analytical gender perspective that goes beyond gender-differentiated reporting along a binary sex/gender variable and considers further categories of social differentiation at the intersection of sex/gender can enable a more differentiated consideration of gender inequalities in GBE. It offers the opportunity to better illustrate developments in the understanding of gender and gender roles as well as the diversity between and within gender groups. Finally, gender-sensitive GBE also has an impact on prevention, health promotion and (health) policy measures in the sense of a *Health in All Policies* strategy and can contribute to greater gender equality in all policy areas.

FUNDING

This pilot project is part of the *Joint Action Prevent Non-Communicable Diseases* (GA - 101128023), which is co-funded by the European Union. Views and opinions expressed are however those of the author(s) only and do not necessarily reflect those of the European Union or European Health and Digital Executive Agency (HaDEA). Neither the European Union nor HaDEA can be held responsible for them.

COMPLIANCE WITH ETHICAL GUIDELINES

Conflict of interest: H. Gencer, A.-C. Saß and F. Prütz declare that there is no conflict of interest.

No studies on humans or animals were carried out by the authors for this article.

REFERENCES

1. Krieger N (2003) Genders, sexes, and health: what are the connections--and why does it matter? Int J Epidemiol 32:652-657. 10.1093/ije/dyg156
2. Robert Koch-Institut (ed) (2020) Gesundheitliche Lage der Frauen in Deutschland. Gesundheitsberichterstattung des Bundes. Gemeinsam getragen von RKI und Destatis. RKI, Berlin
3. Robert Koch-Institut (ed) (2014) Gesundheitliche Lage der Männer in Deutschland. Beiträge zur Gesundheitsberichterstattung des Bundes. RKI, Berlin
4. OECD, Organization for Economic Cooperation and Development (ed) (2023) SIGI 2023 Global Report: Gender Equality in Times of Crisis. Social Institutions and Gender Index. OECD Publishing, Paris
5. Lin V, L'Orange H, Silburn K (2007) Gender-sensitive indicators: Uses and relevance. International Journal of Public Health 52:S27-S34. 10.1007/s00038-006-6049-7
6. Pöge K, Rommel A, Mena E, Holmberg C, Saß A-C, Bolte G (2019) AdvanceGender – Verbundprojekt für eine geschlechtersensible und intersektionale Forschung und Gesundheitsberichterstattung. Bundesgesundheitsbl 62:102-107. 10.1007/s00103-018-2855-3
7. Sen G, Östlin P, George A (2007) Unequal, Unfair, Ineffective and Inefficient Gender Inequity in Health: Why it exists and how we can change it. Final Report to the WHO Commission on Social Determinants of Health. September 2007. Women and Gender Equity Knowledge Network. In:https://eurohealth.ie/wp-content/uploads/2012/02/Unequal-Unfair-Ineffective-and-Inefficient-Gender-Inequity-in-Health.pdf. Accessed: 06.06.2024
8. Röding D, Gerlich MG, Walter U (2024) Gesundheitsindikatoren. In: BzgA, Bundeszentrale für gesundheitliche Aufklärung (ed) Leitbegriffe der Gesundheitsförderung und Prävention. Glossar zu Konzepten, Strategien und Methoden. https://leitbegriffe.bzga.de/alphabetisches-verzeichnis/gesundheitsindikatoren/. Accessed: 04.09.2024
9. Starker A, Rommel A, Saß A-C (2016) Bericht zur gesundheitlichen Lage der Männer in Deutschland – Fazit und Herausforderungen für eine gendersensible Gesundheitsberichterstattung. Bundesgesundheitsbl 59:979-985. 10.1007/s00103-016-2383-y
10. Pöge K, Rommel A, Starker A et al. (2022) Survey of sex/gender diversity in the GEDA 2019/2020-EHIS study – objectives, procedure and experiences. Journal of Health Monitoring 7:48-65. 10.25646/9958
11. Rommel A, Pöge K, Krause L et al. (2019) Geschlecht und Gesundheit in der Gesundheitsberichterstattung des Bundes. Konzepte und neue Herausforderungen. Public Health Forum 27:98-102. 10.1515/pubhef-2019-0021
12. Ziese T, Prütz F, Rommel A, Reitzle L, Saß AC (2020) Gesundheitsberichterstattung des Bundes am Robert Koch-Institut – Status quo und aktuelle Entwicklungen. Bundesgesundheitsbl 63:1057-1066. 10.1007/s00103-020-03195-8
13. Lange C, Lampert T (2004) Perspektiven einer geschlechtersensiblen Gesundheitsberichterstattung. Gesundheitswesen 66:158-163. 10.1055/s-2004-813022
14. Starke D, Tempel G, Butler J, Starker A, Zühlke C, Borrmann B (2019) Gute Praxis Gesundheitsberichterstattung – Leitlinien und Empfehlungen 2.0. Journal of Health Monitoring 4:2-22. 10.25646/6058

15. Robert Koch Institut (ed) (2015) Gesundheit in Deutschland. Gesundheitsberichterstattung des Bundes. Gemeinsam getragen von RKI und Destatis. RKI, Berlin
16. Robert Koch-Institut (ed) (2006) Gesundheit von Frauen und Männern im mittleren Lebensalter. Schwerpunktbericht der Gesundheitsberichterstattung des Bundes. RKI, Berlin
17. GBD 2015 Mortality and Causes of Death Collaborators (2016) Global, regional, and national life expectancy, all-cause mortality, and cause-specific mortality for 249 causes of death, 1980-2015: a systematic analysis for the Global Burden of Disease Study 2015. Lancet 388:1459-1544. 10.1016/s0140-6736(16)31012-1
18. Robert Koch-Institut (ed) (2023) Gesundheitliche Lage der Frauen in Deutschland – wichtige Fakten auf einen Blick. Gesundheitsberichterstattung des Bundes. Gemeinsam getragen von RKI und Destatis. RKI, Berlin
19. Lampert T (2018) Soziale Ungleichheit der Gesundheitschancen und Krankheitsrisiken. Aus Politik und Zeitgeschichte 68:12-18.
20. Lampert T, Kroll LE, Lippe Evd, Müters S, Stolzenberg H (2013) Sozioökonomischer Status und Gesundheit. Bundesgesundheitsbl 56:814-821. 10.1007/s00103-013-1695-4
21. Gößwald A, Schienkiewitz A, Nowossadeck E, Busch M (2013) Prävalenz von Herzinfarkt und koronarer Herzkrankheit bei Erwachsenen im Alter von 40 bis 79 Jahren in Deutschland. Bundesgesundheitsbl 56:650-655. 10.1007/s00103-013-1666-9
22. Busch M, Schienkiewitz A, Nowossadeck E, Gößwald A (2013) Prävalenz des Schlaganfalls bei Erwachsenen im Alter von 40 bis 79 Jahren in Deutschland. 56:656-660. 10.1007/s00103-012-1659-0
23. Hoebel J, Kroll LE, Fiebig J et al. (2018) Socioeconomic Inequalities in Total and Site-Specific Cancer Incidence in Germany: A Population-Based Registry Study. Front Oncol 8:402. 10.3389/fonc.2018.00402
24. Rommel A, Saß A-C, Born S, Ellert U (2015) Die gesundheitliche Lage von Menschen mit Migrationshintergrund und die Bedeutung des sozioökonomischen Status. Bundesgesundheitsbl 58:543-552. 10.1007/s00103-015-2145-2
25. Destatis, Statistisches Bundesamt (ed) (2018) Fragen zur Gesundheit. Rauchgewohnheiten der Bevölkerung 2017. Mikrozensus 2017. Statistisches Bundesamst (Destatis), Wiesbaden
26. WHO, World Health Organization, UNAIDS, Joint United Nations Programme on HIV/AIDS (eds) (2016) A tool for strengthening gender-sensitive national HIV and Sexual and Reproductive Health (SRH) monitoring and evaluation systems. WHO, World Health Organization, Geneva
27. Bolte G (2016) Gender in der Epidemiologie im Spannungsfeld zwischen Biomedizin und Geschlechterforschung. Konzeptionelle Ansätze und methodische Diskussionen. In: Hornberg C, Pauli A, Wede B (eds) Gesundheit – Geschlecht: Eine gesundheitswissenschaftliche Perspektive. Springer VS, Wiesbaden, p 103-124
28. EIGE, European Institute for Gender Equality (ed) (2019) Gender analysis. Gender mainstreaming. Publications Office of the European Union, Luxembourg
29. WHO, World Health Organization (ed) (2020) Incorporating Intersectional Gender Analysis into Research on Infectious Diseases of Poverty: A Toolkit for Health Researchers. World Health Organization, Geneva
30. Mena E, Stahlmann K, Telkmann K, Bolte G, Group obotAS (2023) Intersectionality-Informed Sex/Gender-Sensitivity in Public Health Monitoring and Reporting (PHMR): A Case Study Assessing Stratification on an "Intersectional Gender-Score" International Journal of Environmental Research and Public Health 20:2220. 10.3390/ijerph20032220

31. Iyer A, Sen G, Östlin P (2008) The intersections of gender and class in health status and health care. Global Public Health 1:13-24. 10.1080/17441690801892174
32. Crenshaw K (1989) Demarginalizing the Intersection of Race and Sex: A Black Feminist Critique of Antidiscrimination Doctrine. In: Feminist Legal Theory. Taylor Francis Group, Abingdon, UK, p 139–167
33. Grube MM, Scheidt-Nave C, Gaertner B et al. (2019) Public-Health-Monitoring für die Altersgruppe 65+ in Deutschland – Auswahl und Definition von Indikatoren. Journal of Health Monitoring 4:93-109. 10.25646/5984
34. Gabrys L, Schmidt C, Heidemann C et al. (2017) Diabetes-Surveillance in Deutschland – Hintergrund, Konzept, Ausblick. 210.17886/rki-gbe-2017-006
